# Caput valgum associated with developmental dysplasia of the hip: management by transphyseal screw fixation

**DOI:** 10.1007/s11832-015-0681-9

**Published:** 2015-09-11

**Authors:** Ian P. Torode, Jeffrey L. Young

**Affiliations:** The Royal Children’s Hospital Melbourne, 50 Flemington Road, Parkville, VIC 3052 Australia; Stanford University Medical Center, 300 Pasteur Dr. Edwards R 105, Stanford, CA 94305-5341 USA

**Keywords:** Developmental dysplasia of the hip, Growth disturbance, Caput valgum, Growth modulation

## Abstract

**Purpose:**

A late finding of some hips treated for developmental dysplasia of the hip (DDH) is a growth disturbance of the lateral proximal femoral physis, which results in caput valgum and possibly osteoarthritis. Current treatment options include complete epiphysiodesis of the proximal femoral physis or a corrective proximal femoral osteotomy. Alternatively, a transphyseal screw through the inferomedial proximal femoral physis that preserves superolateral growth might improve this deformity.

**Methods:**

This study evaluates the effect of such a transphyseal screw on both femoral and acetabular development in patients with caput valgum following open treatment of DDH. These patients were followed clinically and radiographically until skeletal maturity. Preoperative and postoperative radiographs were assessed, measuring the proximal femoral physeal orientation (PFPO), the head–shaft angle (HSA), Sharp’s angle and the center edge angle of Wiberg (CE angle).

**Results:**

Thirteen hips of 11 consecutive patients were followed prospectively. The age at the time of transphyseal screw placement was between 5 and 14 years. The mean improvement of the PFPO and HSA was 14° (*p* < 0.01) and 11° (*p* < 0.001), respectively. The mean improvement of Sharp’s angle and CE angle was 4.7° (*p* < 0.01) and 5.8° (*p* < 0.02), respectively. Five patients underwent screw revision.

**Conclusions:**

A transphyseal screw across the proximal femoral physis improved the proximal femur and acetabular geometry.

## Introduction

**Fig. 1:**
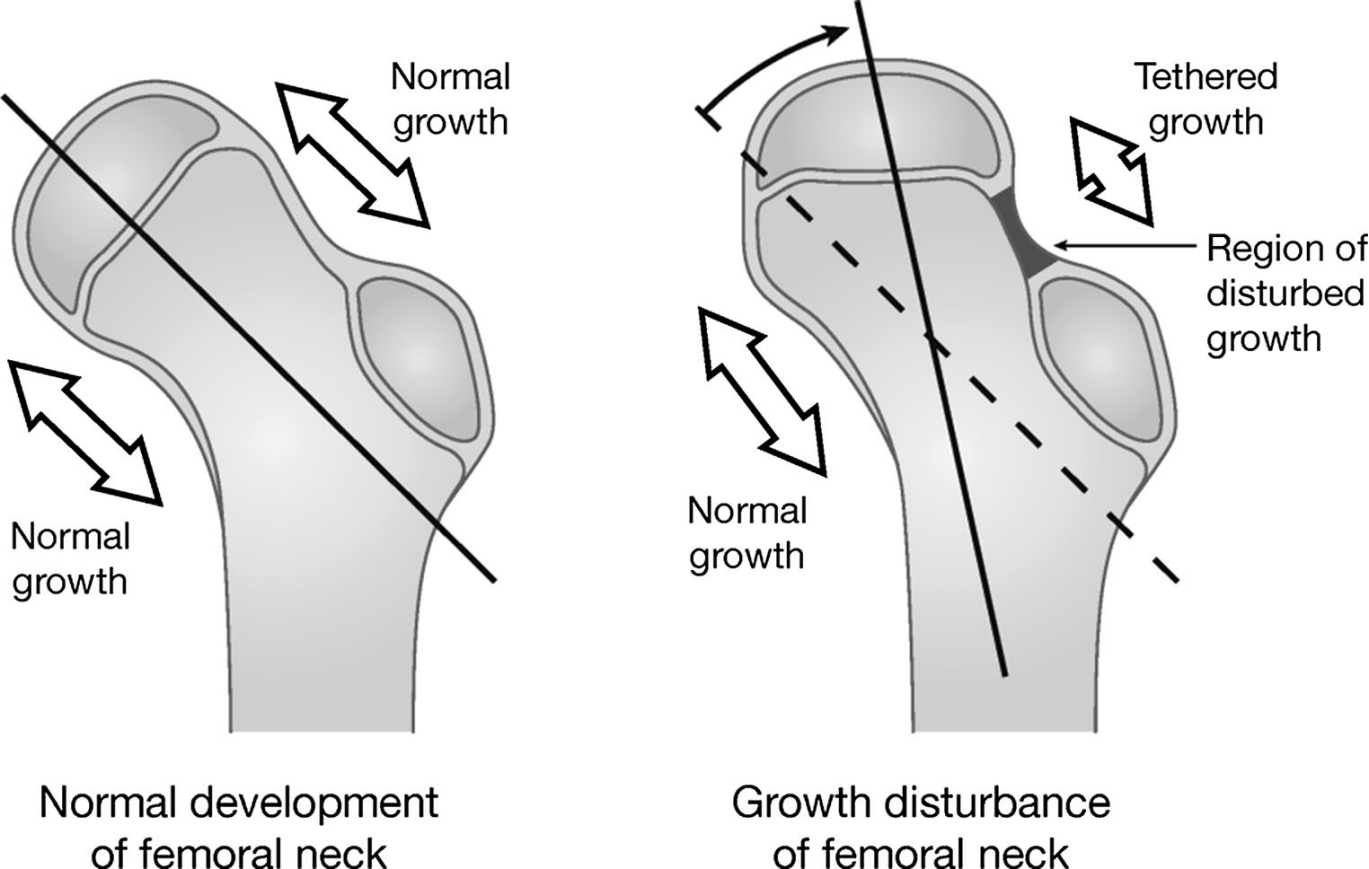
The most common growth disturbance following treatment of developmental dysplasia of the hip occurred in the lateral aspect of the proximal femoral physis. This growth disturbance resulted in a horizontal orientation of the proximal femoral physis and caput valgum deformity

Several studies have described deformities of the femoral head and neck that arise from growth disturbance of the proximal femoral physis following treatment of developmental dysplasia of the hip (DDH) [1–5]. In a study by Kalamchi and MacEwen, the most common pattern of growth disturbance following treatment of developmental dysplasia occurred in the lateral proximal femoral physis [4]. As a result of this growth disturbance, the proximal femoral physis became horizontally oriented and the femoral head appeared in valgus relative to the femoral neck, resulting in a caput valgum deformity (Fig. 1). Ogden suggested that this growth disturbance resulted from occlusion by compression of the lateral epiphyseal branch of the medial circumflex artery in either the superior or posterior intra-epiphyseal groove by the acetabular labrum [6]. This is consistent with the findings by others that excessive abduction is a risk factor of growth disturbance in DDH [1–5].

The sequelae of caput valgum deformity are unpredictable, but may result in poor acetabular development, acetabular dysplasia and early degenerative arthritis [3, 4, 7, 8]. Early recognition of deformity is difficult, and late management is challenging. When recognized early, a complete epiphysiodesis of the proximal femoral physis would maintain the femoral–acetabular relationship, but at the expense of femoral neck length. When recognition occurs after the development of a caput valgum deformity, an acute corrective osteotomy might be considered. However, the osteotomy is often performed at the level of the lesser trochanter and introduces a translational deformity. Another disadvantage is that a corrective osteotomy performed prior to skeletal maturity incurs the risk of recurrent deformity, if the growth disturbance of the proximal femoral physis remains untreated.

With recent studies evaluating guided growth in skeletally immature children [9–11], another option for treating caput valgum is to insert a transphyseal screw through the inferomedial proximal femoral physis. The hypothesis is that gradual correction of the caput valgum occurs. This paper continues the follow-up of early results presented previously [12]. That study reported the early radiographic changes in the proximal femur and acetabulum following the insertion of a transphyseal screw for the treatment of caput valgum associated with DDH.

## Materials and methods

Between 2003 and 2007, consecutive patients treated with a transphyseal screw for caput valgum were prospectively followed. All patients were undergoing ongoing clinical and radiographic review following open treatment via a medial approach for DDH. The senior author selected patients for transphyseal screw placement when increasing caput valgum deformity was recognized on serial radiographs. Patients with hip dysplasia secondary to neuromuscular conditions, arthrogryposis, or other teratologic conditions were excluded.

**Fig. 2:**
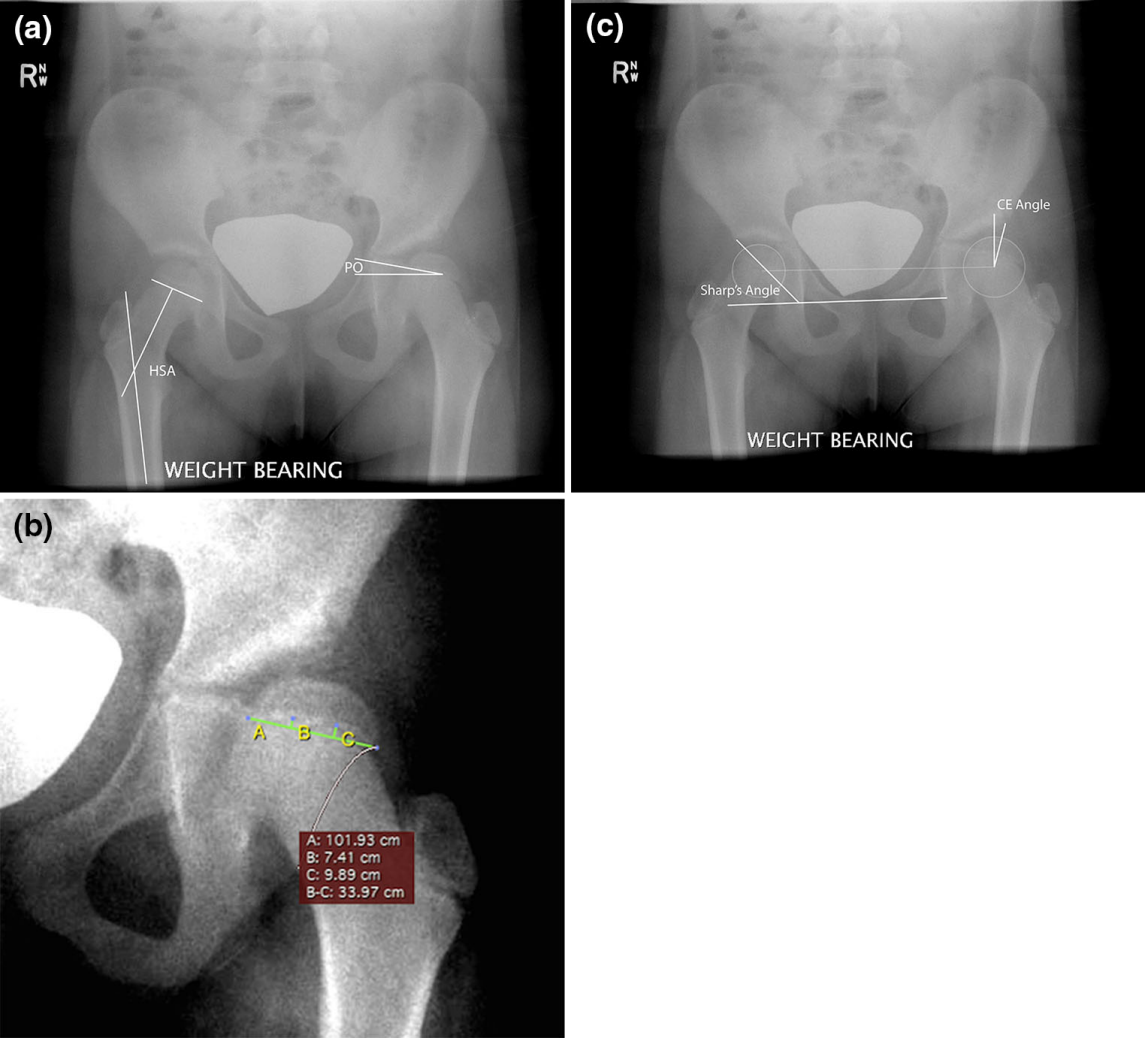
Radiographic measurements: **a** the head–shaft angle (HSA) is the angle between a line perpendicular to the proximal femoral physis and the femoral shaft. The physeal orientation (PO) is the angle between the horizon and the line through the middle 1/3 of the proximal femoral physis. **b** The middle 1/3 of the proximal femoral physis was defined by a line connecting the medial point (*point B*) and lateral point (*point C*) of the middle 1/3 of the physis. **c** Sharp’s angle is measured from the radiographic teardrop to the lateral margin of the acetabulum and a line connecting both teardrops. The center edge angle of Wiberg (CE) is measured from the center of the femoral head to the lateral margin of the acetabulum and a line perpendicular to a line connecting the center of both femoral heads

Each patient had serial radiographs of the pelvis every 6 months until skeletal maturity. Radiographs of the pelvis were standardized by imaging patients standing, with patellae forward. After skeletal maturity, children were followed annually. From these pelvic radiographs, the proximal femur geometry was measured by measuring the physeal orientation and the head–shaft angle, as shown in Fig. 2a. The physeal orientation was defined as the angle between the middle 1/3 of the proximal femoral physis and a horizontal line. Since the proximal femoral physis is curvilinear, the middle 1/3 was defined by a line that connected the medial and lateral points of the middle 1/3 of the physis (Fig. 2a, b). The physeal orientation was recorded as positive when the physis faced toward the acetabulum, and negative when the physis faced away from the acetabulum. The head–shaft angle was measured by the technique reported by Foroohar et al. [13] and previously by Southwick [14]. Acetabular changes were measured by Sharp’s angle [15] and the center edge angle of Wiberg (CE angle) [16], as shown in Fig. 2c. Measurements were performed using OsiriX (Pixmeo SARL, Bernex, Switzerland). Preoperative and postoperative radiographic angles were analyzed using paired *t*-test statistics with SPSS^®^ (International Business Machines Corporation, Amonk, NY, USA). Treated and untreated extremities were analyzed using Student’s *t* test.

Since the physeal orientation measurement is a novel measurement, intra- and inter-rater reliability was tested using the interclass correlation (ICC). Intra-rater reliability was assessed by repeating preoperative physeal orientation measurements with more than 1 year between measurements. Inter-rater reliability was assessed by comparing preoperative physeal orientation measurements performed by a pediatric orthopaedic surgeon and performed by a pediatric radiologist.

In addition to the above measurements, complications were noted, including infection and symptomatic hardware. Complications also included the epiphysis growing off the screw. When this was noted, revision surgery was performed by either advancing the existing screw or replacing the screw with a longer screw.

### Surgical technique

Patients were positioned supine on a radiolucent operating table. After routine preparation, the affected limb was draped to allow free movement of the hip. Under the image intensifier, the hip was examined and positioned, usually rotated internally, to show the maximum profile of the femoral neck. Through a 2-cm incision, a guide wire was introduced to enter the lateral and proximal aspect of the femur just distal to the greater trochanteric growth plate. The guide wire was passed across the inferomedial half of the proximal femoral physis. Care was taken to avoid advancing the guide wire into the hip joint. The length of screw was measured off the guide wire, with the expectation that the screw should be slightly proud on the lateral aspect of the femur. The screw was then passed carefully over the guide wire into the femoral head. At the stage where the screw advances on the threads of the guide wire, additional caution was exercised to avoid inadvertently advancing the wire into and across the hip. The screw was advanced until the tip of the screw reached the subchondral bone, and the position was confirmed with the image intensifier. In this series an 8-mm Stryker ASNIS (Stryker Orthopaedics, Mahwah, NJ, USA) or a 7.3-mm Synthes cannulated screw (Synthes, Oberdorf, Switzerland) was used. Fully threaded screws were preferred to facilitate potential screw removal later, but partially threaded screws were used when fully threaded screws were unavailable.

## Results

Thirteen hips in 11 patients were evaluated. Demographics are summarized in Table 1. Eight patients were females, and three were males. Nine patients had unilateral involvement, and two patients had bilateral involvement. The age of patients at the time of transphyseal screw insertion averaged 9 years 3 months (range 5 years 8 months–14 years 3 months). Mean follow-up time was 60 months (range 19–104 months). All hips were followed until skeletal maturity.

**Table 1:** Demographics

Patient	Gender	Age at time of screw insertion (years)	Side	Follow-up (months)
1	F	8	R	70
2	F	8	R	21
3	F	5	R	81
4	M	12	R	37
5	M	10	R	70
6	M	14	L	19
7	F	7	L	75
8	F	12	L	80
9	F	8	L	73
10	F	7	B	104
11	F	11	B	24

**Fig. 3:**
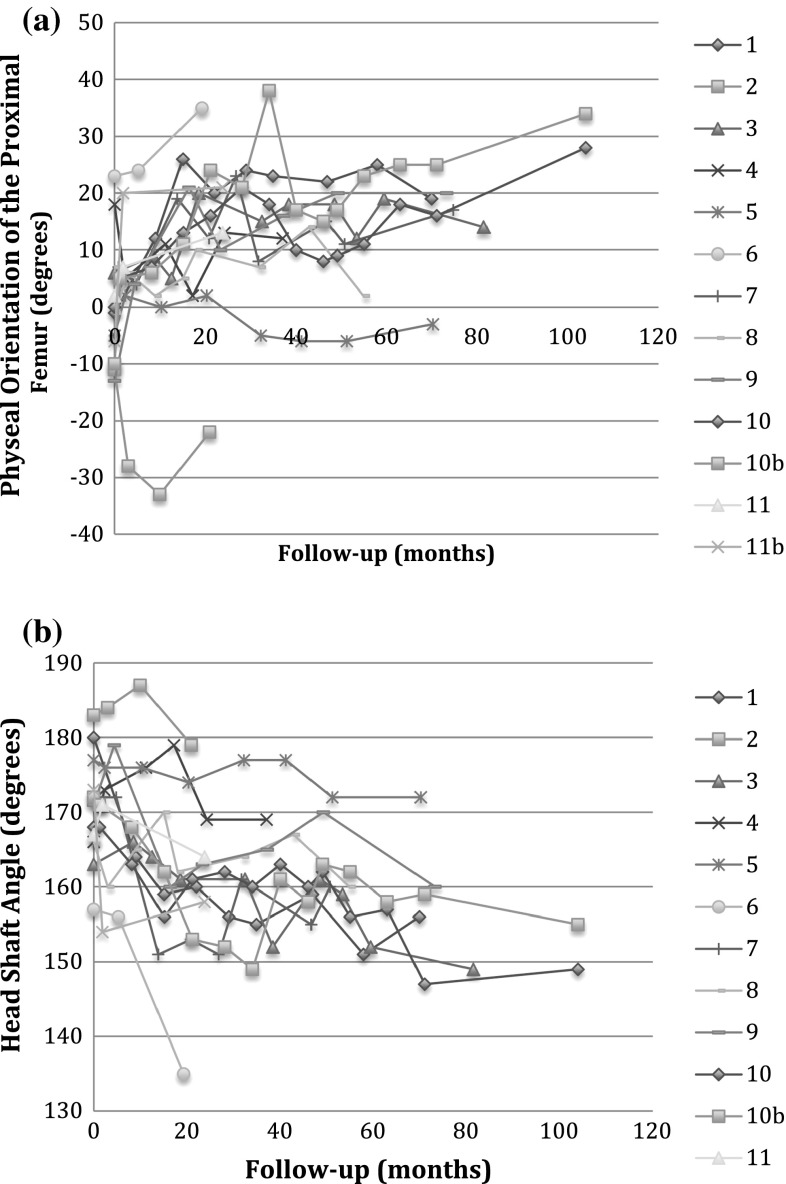
**a** Physeal orientation of the proximal femur of hips treated with transphyseal screws versus follow-up time. **b** Head–shaft angle of hips treated with transphyseal screws versus follow-up time

Hips treated with the transphyseal screw had radiographic improvements in the orientation of the proximal femoral physis (Table 2; Fig. 3). The physeal orientation of the proximal femur of treated hips improved by a mean of 14° ± 16° (mean ± standard deviation), with a 95 % confidence interval (CI) of 4.2°–24° (*p* = 0.009). These hips had a mean preoperative physeal orientation of 0.4° ± 11°, and a mean final physeal orientation of 15° ± 16°. By comparison, the untreated hips showed no significant changes: the mean preoperative physeal orientation measured 14° ± 3.6°, and the mean final physeal orientation measured 12° ± 7.8° (*p* = 0.61). At final follow-up, the physeal orientations of treated and untreated hips were not statistically different (*p* = 0.7). The ICC for intra- and inter-rater reliability was 0.91 (95 % CI = 0.79–0.96) and 0.89 (95 % CI = 0.74–0.95), respectively. The head–shaft angle of treated hips improved by a mean of 11 ± 8.1° (95 % CI = 6.6°–16°, *p* < 0.001). These hips had a mean preoperative head–shaft angle of 170° ± 7.0°, and a mean final head–shaft angle of 159° ± 11°. By comparison, the uninvolved hips showed no significant changes: the mean preoperative head–shaft angle measured 160° ± 4.1°, and the mean final head–shaft angle measured 159° ± 7.2° (*p* = 0.82). At final follow-up, the head–shaft angles of treated and untreated hips were not statistically different (*p* = 0.9).

**Table 2:** Radiographic measurements—proximal femur

Patient	Preoperative physeal orientation—untreated side (°)	Preoperative physeal orientation—treated side (°)	Postoperative physeal orientation—untreated side (°)	Postoperative physeal orientation—treated side (°)	Preoperative HSA—untreated side (°)	Preoperative HSA—treated side (°)	Postoperative HSA—untreated side (°)	Postoperative HSA—treated side (°)
1	15	−1	22	19	158	180	158	156
2	11	−11	9	−22	167	183	161	179
3	14	6	7	14	158	163	151	149
4	19	18	3	12	159	166	172	169
5	9	−5	9	−3	163	177	169	172
6	9	23	10	35	159	157	153	135
7	14	−5	7	17	161	172	153	160
8	18	7	23	2	152	166	157	160
9	16	−13	23	20	161	171	159	160
10		0		28		168		149
		−10		34		172		155
11		2		13		167		164
		−6		21		173		158

*HSA* Head–shaft angle

**Fig. 4:**
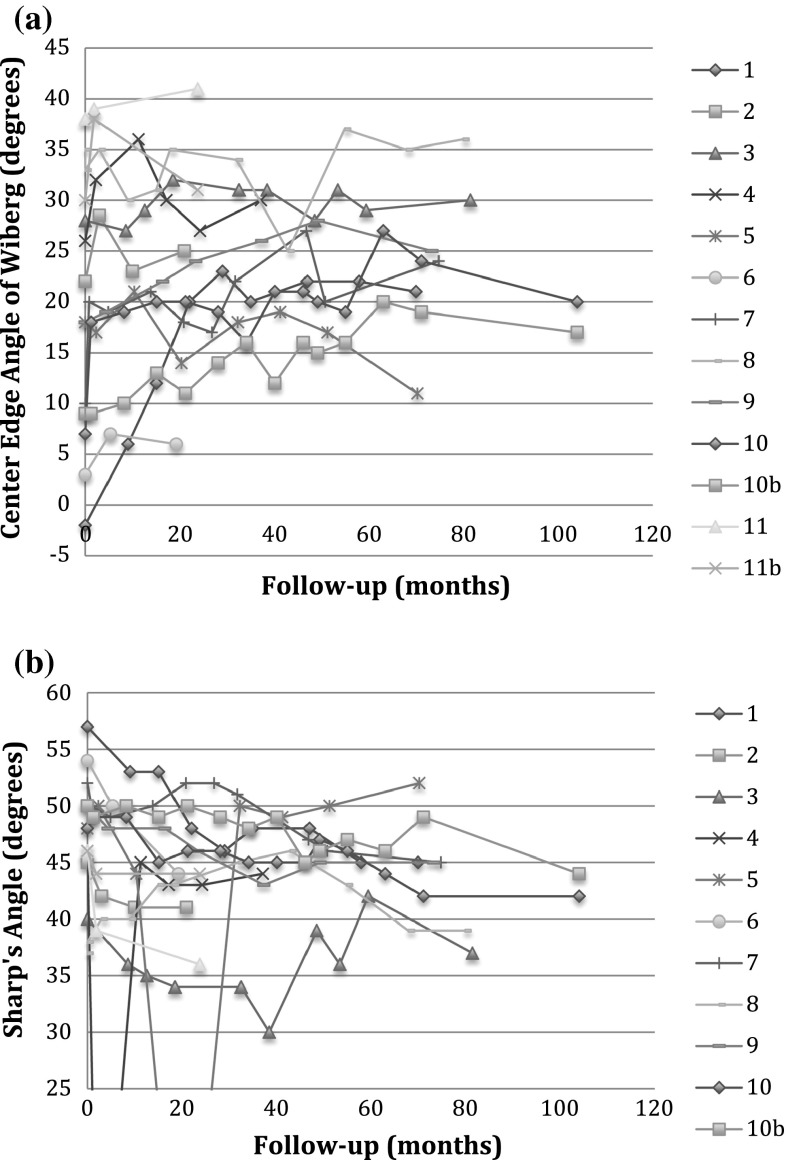
**a** Center edge angle of Wiberg of hips treated with transphyseal screws versus follow-up time. **b** Sharp’s angle of hips treated with transphyseal screws versus follow-up time

Acetabular measurements also showed improvements in affected hips treated with a transphyseal screw (Table 3; Fig. 4). Sharp’s angle of treated hips improved by a mean of 4.7° ± 4.6° (95 % CI = 1.9°–7.5°, *p* = 0.003). These hips had a mean preoperative Sharp’s angle of 47° ± 5.3°, and a mean final Sharp’s angle of 43° ± 4.1°. By comparison, Sharp’s angle for the untreated hip did not change: the mean preoperative Sharp’s angle measured 46° ± 2.6°, and the mean final Sharp’s angle measured 44° ± 4.0° (*p* = 0.15). The CE angle of treated hips improved by a mean of 5.8° ± 7.5° (95 % CI = 1.2°–10°, *p* = 0.017). These hips had a mean preoperative CE angle of 19° ± 13° and a mean final CE angle of 24° ± 9.7°. By comparison, the CE angle for the untreated hip showed no improvement: the mean preoperative CE angle measured 28° ± 7.9°, and the mean CE angle at final follow-up measured 28° ± 7.0°. At skeletal maturity, treated hips and uninvolved hips were not statistically different (*p* = 0.08).

**Table 3:** Radiographic measurements—acetabulum

Patient	Preoperative Sharp’s angle—untreated side (°)	Preoperative Sharp’s angle—treated side (°)	Postoperative Sharp’s angle—untreated side (°)	Postoperative Sharp’s angle—treated side (°)	Preoperative CE angle—untreated side (°)	Preoperative CE angle—treated side (°)	Postoperative CE angle—untreated side (°)	Postoperative CE angle—treated side (°)
1	45	57	36	45	30	−2	37	21
2	46	45	44	41	25	22	26	25
3	47	40	47	37	20	28	24	30
4	47	46	42	44	29	26	25	30
5	51	48	47	52	19	18	23	11
6	49	54	50	44	24	3	20	6
7	47	52	44	45	26	10	28	24
8	43	37	43	39	42	35	42	36
9	43	50	47	45	39	18	30	25
10		48		42		7		20
		50		44		9		17
11		46		36		38		41
		46		44		30		31

*CE* center edge

No early complications were noted in this series. There were screw revisions in five hips in four patients, occurring 25–49 months following insertion of the transphyseal screw. In each case, the screw was advanced or exchanged for a longer screw to cross the physis. The transphyseal screw was removed in two patients following skeletal maturity upon each patient’s request. One patient underwent trochanteric advancement at the time of transphyseal screw removal.

## Discussion

**Fig. 5:**
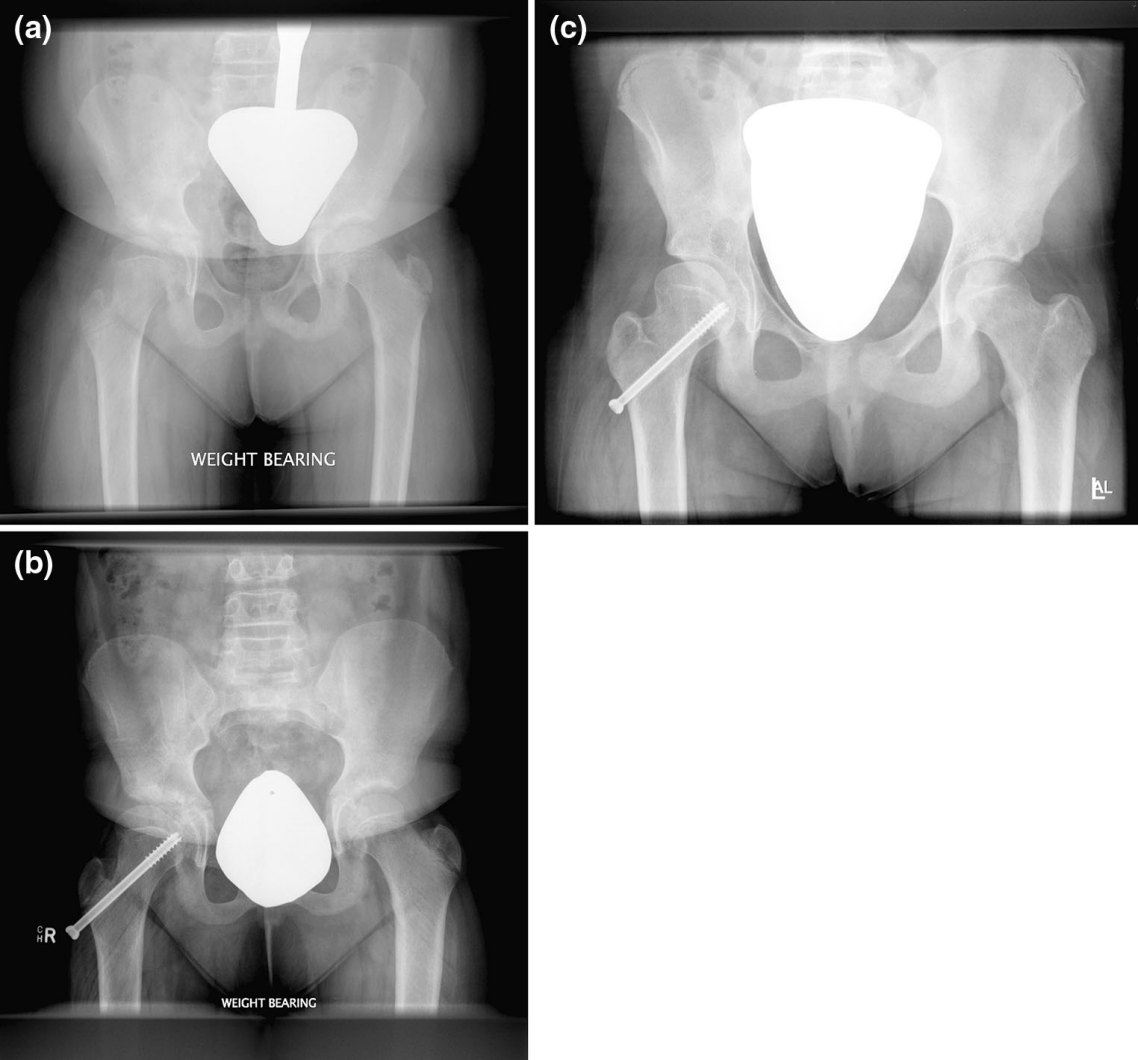
**a** Preoperative radiograph of an 8-year-old patient with right DDH. **b** Pelvic radiograph following insertion of a right transphyseal screw. **c** Pelvic radiograph 5 years after the insertion of the transphyseal screw

The purpose of this investigation was to study the effects of a transphyseal screw on the growth of the proximal femoral physis in patients with caput valgum. Metaizeau et al. described the use of transphyseal screw for angular correction in the knee [10], and Stevens et al. described the use of a medial malleolar screw for angular correction of ankle valgus [11]. Use of a transphyseal screw has been shown in animal studies to cause angular change in the proximal femoral physis [17]. This study evaluated the use of a transphyseal screw in the setting of a growth disturbance of the proximal femoral physis that resulted in a caput valgum deformity, Fig. 5. Similar to other studies, the age range of our patients at the time of initial identification was 5–14 years [4, 5, 8, 18]. Though some patients approached skeletal maturity, all patients had open physes. Transphyseal screw insertion was performed with the intention that some correction is better than no correction or progressive caput valgum deformity. At skeletal maturity, significant improvement occurred in the orientation of the proximal femoral physis, as measured by the physeal orientation and the head–shaft angle. Following transphyseal screw placement, the physeal orientation improved by a mean of 14° and the head–shaft angle improved by a mean of 11°. By comparison, untreated hips in patients with unilateral transphyseal screw placement showed no significant change in physeal orientation or head–shaft angle during this study.

Defining the geometry of the proximal femur on two-dimensional radiographs has limitations as compared with three-dimensional imaging modalities. Serial computer tomography would expose patients to unacceptably high levels of radiation and serial magnetic resonance imaging is cost-prohibitive. On plain film radiographs, abduction and adduction of the hip, as well as internal and external rotation of the hip, can affect radiographic measurements. As such, efforts were made to standardize the position of the patient, obtaining radiographs with the patient standing, patellae forward, and hips with neutral abduction/adduction. Prior work by Foroohar et al. showed that the head–shaft angle had good inter-rater reliability and minimal variability due to rotation [13]. Since the orientation of the physis was made relative to the femoral shaft, one disadvantage of the head–shaft angle measurement is that it does not differentiate between coxa valga and caput valgum, valgus orientation of the femoral neck and head, respectively.

The physeal orientation was measured intending to separate caput valgum from coxa valga by disregarding the neck shaft angle. The orientation of the middle 1/3 was measured, as opposed to the medial and lateral edges, because following growth disturbance of the physis, the physis is often nonlinear. The physeal orientation was measured with respect to the ground and not the femoral neck, because the femoral neck in patients with caput valgum is short and accurate measurement is challenging. McGillion and Clarke referenced the orientation of the proximal femoral physis to the pelvis when they measured “tilt angle” [19]. Measuring the orientation of the physis to the ground, however, eliminates variability due pelvic tilt. The physeal orientation measurements showed good intra- and inter-rater reliability.

A secondary change noted in patients treated with transphyseal screw fixation of the proximal femur was an improvement in acetabular geometry. Sharp’s angle improved by 4.7° and the center edge angle improved by 5.8°. The acetabular index was not measured in this study because it becomes increasingly difficult to measure as the triradiate cartilage closes when patients become skeletally mature. Meanwhile, Sharp’s angle references landmarks present through development, and it shows little variation with pelvic tilt and pelvic rotation [15]. Furthermore, prior studies report good inter- and intra-rater reliability for Sharp’s angle and center edge angle of Wiberg [20]. While the mean changes in Sharp’s angle and the CE angle were small, in some children the improvement was remarkable. In reviewing and measuring many radiographs, it appears that the central third of the proximal femoral physis is relatively straight, while the superior and inferior margins droop. This central third of the physis corresponds to the roundest part of the femoral head, which could be a stimulus for acetabular improvement. However, this is difficult to document but deserves further investigation.

At skeletal maturity, hips with caput valgum deformity treated with transphyseal screw compared to the contralateral hip in patients with unilateral deformity were not statistically different. These results support the hypothesis that placement of a transphyseal screw induces beneficial changes to the proximal femur, as well as the acetabulum. However, in comparison with published normal values, the head–shaft angle, the center edge angle of Wiberg, and Sharp’s angle for hips at skeletal maturity in this case series were in the upper limits of normal, or slightly worse than normal. Foroohar et al. reported the normal values of the head–shaft angle in typically developing children to be 152.5° [13]. The head–shaft angle of patients in this study at skeletal maturity was more similar to hips in patients with cerebral palsy not warranting surgical intervention. The mean final Sharp’s angle of treated hips and untreated hips in this series was 43° and 44°, respectively. This was slightly greater than the 42° reported by Sharp to be the upper limit of normal [15]. Lastly, the CE angle of patients in this series measured 24° and 28° for untreated hips and treated hips at skeletal maturity, respectively, resulting in borderline normal values of the CE angle [16, 21]. Comparing the treated and untreated hips to normal values suggests that even untreated hips had subtle abnormalities and longer-term follow-up is warranted.

**Fig. 6:**
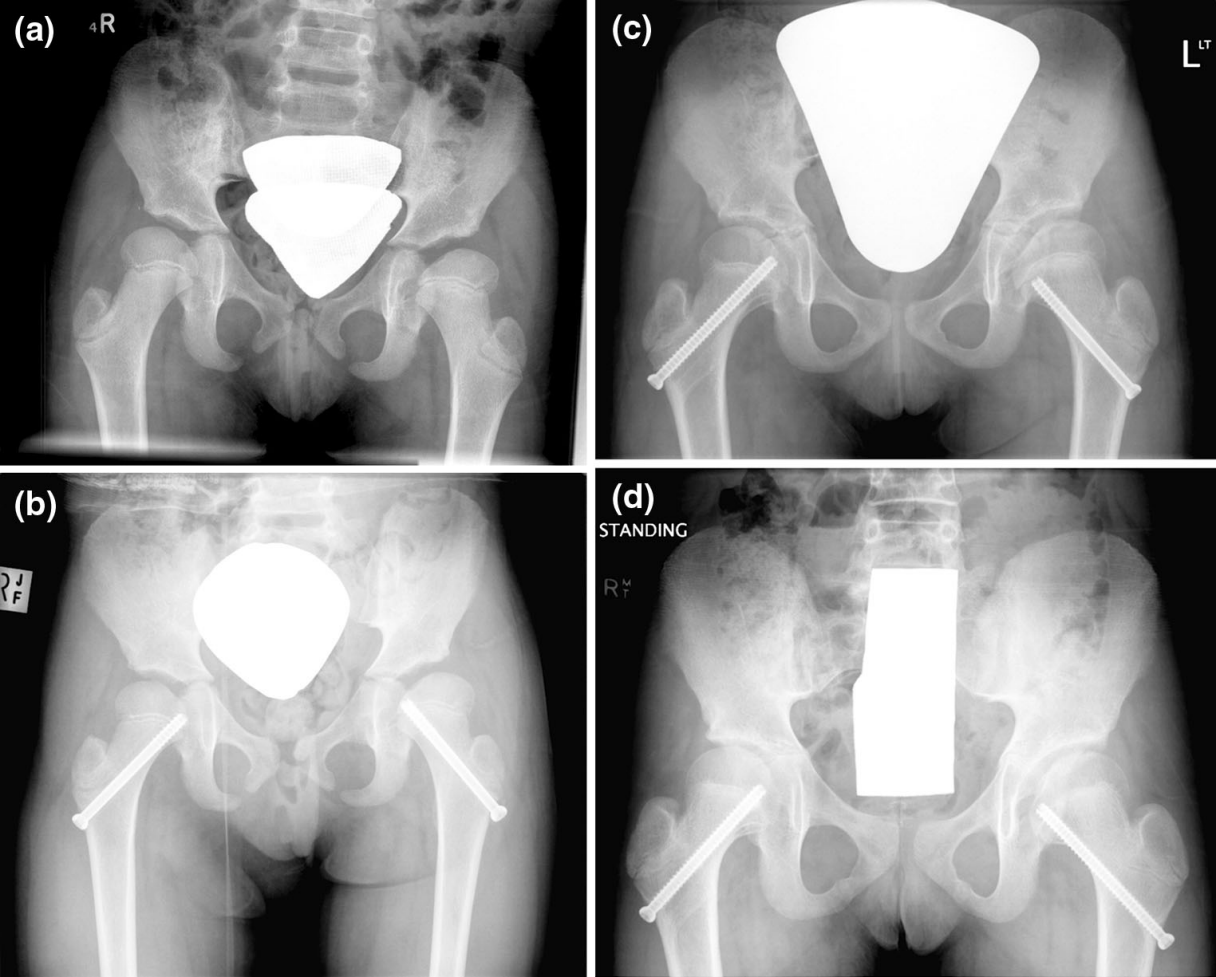
**a** Preoperative radiograph of a 7-year-old patient with bilateral DDH. **b** Pelvic radiograph following insertion of bilateral transphyseal screws. **c** Pelvic radiograph 4 years after insertion; the right transphyseal screw was revised and the left transphyseal screw requires revision. **d** Pelvic radiograph 5 years 6 months after the index insertion of bilateral transphyseal screws

The most common complication in this study was the proximal femoral epiphysis growing off the screw necessitating screw revision; see Fig. 6. With the epiphysis being small, only a limited number of screw threads would engage the femoral head epiphysis. Carney et al. showed progressive slippage in patients with slipped capital femoral epiphysis when less than five threads engaged the proximal femoral epiphysis [22]. This group of patients requiring revision of the transphyseal screw to recapture the proximal femoral physis still made improvements in head–shaft angle and physeal orientation. The small group size, however, limits analysis. This series includes patients with partially threaded screws and fully threaded screws, based on available hardware at the time of surgery. The senior author’s preference is for fully threaded screws to facilitate removal; however, to date, there has been no difficulty in removing partially threaded screws when needed. One patient underwent trochanteric advancement to address the short femoral neck resulting from the lateral growth disturbance.

In summary, early identification of a lateral growth arrest of the capital femoral epiphysis may be treated with application of a transphyseal screw. The improvement in the physeal orientation and head–shaft angle reflects the effectiveness of guided growth principles in the proximal femur. Furthermore, the improvement of the femoral head may impart beneficial effects to the acetabulum as well, though further investigation is required.

## References

[1] Brougham DI (1990). Avascular necrosis following closed reduction of congenital dislocation of the hip. Review of influencing factors and long-term follow-up. J Bone Joint Surg Br.

[2] Bucholtz RW, Ogden JA (1978) Patterns of ischemic necrosis of the proximal femur—nonoperatively treated congenital hip disease. In: The hip: proceedings of the sixth open scientific meeting of the hip society. C.V. Mosby, St. Louis

[3] Cooperman DR, Wallensten R, Stulberg SD (1980). Post-reduction avascular necrosis in congenital dislocation of the hip. J Bone Joint Surg Am.

[4] Kalamchi A, MacEwen GD (1980). Avascular necrosis following treatment of congenital dislocation of the hip. J Bone Joint Surg Am.

[5] Campbell P, Tarlow SD (1990). Lateral tethering of the proximal femoral physis complicating the treatment of congenital hip dysplasia. J Pediatr Orthop.

[6] Ogden JA (1974) Anatomic and histologic study of factors affecting development and evolution of avascular necrosis in congenital dislocation of the hip. In: William H. Harris (ed) The hip: proceedings of the second open scientific meeting of the hip society. C.V. Mosby, St. Louis

[7] Oh CW (2009). A radiological classification of lateral growth arrest of the proximal femoral physis after treatment for developmental dysplasia of the hip. J Pediatr Orthop.

[8] Kim HW (2000). Acetabular development in developmental dysplasia of the hip complicated by lateral growth disturbance of the capital femoral epiphysis. J Bone Joint Surg Am.

[9] Stevens PM, Klatt JB (2008). Guided growth for pathological physes: radiographic improvement during realignment. J Pediatr Orthop.

[10] Metaizeau JP (1998). Percutaneous epiphysiodesis using transphyseal screws (PETS). J Pediatr Orthop.

[11] Stevens PM, Belle RM (1997). Screw epiphysiodesis for ankle valgus. J Pediatr Orthop.

[12] Torode IP (2010) Coxa valga associated with developmental dysplasia of the hip: management with a transphyseal screw. In: Pediatric Orthopaedic Society of North America (POSNA) annual meeting 2010, Waikoloa, Hawaii

[13] Foroohar A (2009). Head-shaft angle measurement in children with cerebral palsy. J Pediatr Orthop.

[14] Southwick WO (1967). Osteotomy through the lesser trochanter for slipped capital femoral epiphysis. J Bone Joint Surg Am.

[15] Sharp IK (1961). Acetabular dysplasia. J Bone Joint Surg Br.

[16] Wiberg G (1939). Studies on dysplastic acetabula and congenital subluxation of the hip joint. Acta Chir Scand.

[17] McCarthy JJ (2010). Guided growth of the proximal femur: a pilot study in the lamb model. J Pediatr Orthop.

[18] Morcuende JA (1997). Long-term outcome after open reduction through an anteromedial approach for congenital dislocation of the hip. J Bone Joint Surg Am.

[19] McGillion S, Clarke NM (2011). Lateral growth arrest of the proximal femoral physis: a new technique for serial radiological observation. J Child Orthop.

[20] Engesaeter IO (2012). Radiological findings for hip dysplasia at skeletal maturity. Validation of digital and manual measurement techniques. Skelet Radiol.

[21] Fredensborg N (1976). The CE angle of normal hips. Acta Orthop Scand.

[22] Carney BT, Birnbaum P, Minter C (2003). Slip progression after in situ single screw fixation for stable slipped capital femoral epiphysis. J Pediatr Orthop.

